# Single Step Laser-Induced Deposition of Plasmonic Au, Ag, Pt Mono-, Bi- and Tri-Metallic Nanoparticles

**DOI:** 10.3390/nano12010146

**Published:** 2021-12-31

**Authors:** Daria V. Mamonova, Anna A. Vasileva, Yuri V. Petrov, Alexandra V. Koroleva, Denis V. Danilov, Ilya E. Kolesnikov, Gulia I. Bikbaeva, Julien Bachmann, Alina A. Manshina

**Affiliations:** 1 Institute of Chemistry, Saint-Petersburg State University, 26 Universitetskii Prospect, 198504 Saint-Petersburg, Russia; magwi@mail.ru (D.V.M.); anvsilv@gmail.com (A.A.V.); st086467@student.spbu.ru (G.I.B.); julien.bachmann@fau.de (J.B.); 2 Department of Physics, Saint-Petersburg State University, Ulyanovskaya 3, 198504 Saint-Petersburg, Russia; y.petrov@spbu.ru; 3 Center for Physical Methods of Surface Investigation, Research Park, Saint Petersburg University, Universitetskiy Prosp. 35, Lit. A, 198504 Saint-Petersburg, Russia; koroleva.alexandra.22@gmail.com; 4 Interdisciplinary Resource Center for Nanotechnology, Research Park, Saint-Petersburg State University, Ulyanovskaya 1, 198504 Saint-Petersburg, Russia; danilov1denis@gmail.com; 5 Center for Optical and Laser Materials Research, Research Park, Saint-Petersburg State University, Ulyanovskaya 5, 198504 Saint-Petersburg, Russia; ilya.kolesnikov@spbu.ru; 6 Department of Chemistry and Pharmacy, Friedrich–Alexander University of Erlangen–Nürnberg, IZNF, Cauerstr. 3, 91058 Erlangen, Germany

**Keywords:** laser-induced deposition, noble metal NPs, multimetallic nanoparticles, plasmon resonance

## Abstract

Multimetallic plasmonic systems usually have distinct advantages over monometallic nanoparticles due to the peculiarity of the electronic structure appearing in advanced functionality systems, which is of great importance in a variety of applications including catalysis and sensing. Despite several reported techniques, the controllable synthesis of multimetallic plasmonic nanoparticles in soft conditions is still a challenge. Here, mono-, bi- and tri-metallic nanoparticles were successfully obtained as a result of a single step laser-induced deposition approach from monometallic commercially available precursors. The process of nanoparticles formation is starting with photodecomposition of the metal precursor resulting in nucleation and the following growth of the metal phase. The deposited nanoparticles were studied comprehensively with various experimental techniques such as SEM, TEM, EDX, XPS, and UV-VIS absorption spectroscopy. The size of monometallic nanoparticles is strongly dependent on the type of metal: 140–200 nm for Au, 40–60 nm for Ag, 2–3 nm for Pt. Bi- and trimetallic nanoparticles were core-shell structures representing monometallic crystallites surrounded by an alloy of respective metals. The formation of an alloy phase took place between monometallic nanocrystallites of different metals in course of their growth and agglomeration stage.

## 1. Introduction

Plasmonic nanoparticles are a matter of intense current research in many scientific directions connected with the development of synthetic procedures [1,2], various applications in electrochemistry and catalysis [3,4,5], phase contrast in bioimaging [6], and metal-enhanced fluorescence (MEF) [7]. Plasmonic nanoparticles are widely used in surface-enhanced Raman scattering (SERS) [8] and variants of SERS such as spread spectrum SERS (ss-SERS) [9], plasmon-enhanced stimulated Raman scattering (PESRS) microscopy with single-molecule detection sensitivity [10], catalyzing processes with real-time SERS monitoring [11], etc. The key feature of plasmonic NPs appears in the interaction between metal nanoparticles and electromagnetic waves and leads to free electron oscillations known as localized surface plasmon resonance (LSPR). LSPR frequency is strongly sensitive to the material of NPs, its size, shape, morphology, and surrounding medium [12]. By variation of these parameters, the LSPR can be tuned in a wide spectral range and optimized for exact sensing and spectroscopy application [13,14]. The interest in plasmonic NPs is even higher for multimetallic nanosystems such as bi- and trimetallic structures of various morphology and phase composition—alloys, core/shell, dimers, bimodal nanoparticles (Janus structure) etc. Multimetallic systems demonstrate an advantage over monometallic nanoparticles due to the peculiarity of electronic structure appearing in advanced functionality in various catalytic and sensing applications [15,16,17,18,19]. Functional features of multimetallic nanosystems depend strongly on their morphology and nature of the multimetallic phase—a mixture of pure metals, or alloy, or core/shell structures. The main factors determining the kind of multimetallic phase are connected with the chemical nature of metals and their phase diagrams. Another very important factor is the synthetic process that can favor the formation of one or the other phase kind dependent on synthesis conditions. I.e Tang et al. [20] reported the formation of AgPt, AgPd, and CuPt alloy nanoparticles and AgCuPt core-shell structure with an Ag core and CuPt shell by a co-reduction method. Selective incorporation of Pt on the Ag/Au nanoparticles by successive reduction method was demonstrated by Thongthai et al. [21]. AuCu/Pt trimetallic core-shell nanoparticles were synthesized with a one-pot synthesis method [22]. Core-shell structures that are overlapping crystallites of Au and Pt metals into a thin layer of Ag nanoparticles as a shell were demonstrated by the microwave synthesis method [23]. Zhou et al. [24] used a seed-mediated growth process and demonstrated morphology of trimetallic Pt-Au-Ag NPs that is Pt shell and Au/Ag core.

Despite a rather deep elaboration of a theory of solid solubility and detailed description of phase diagrams and thermodynamic properties of metals [25,26], scientists meet new challenges when studying these processes at the nanoscale and for nanostructures. That is why the accumulation of knowledge about the nanoscale structure of multimetallic nanosystems obtained by different experimental techniques will provide progress in the understanding of the factors governing the occurrence of the alloy nanophases of different metals in various synthesis conditions.

Here we present synthesis of mono and multimetallic (bi- and tri-metallic) nanoparticles obtained by laser-induced deposition (LID) from solutions of precursors of metals (salts, organometallic complexes) [27,28]. The peculiarity of the LID process is the formation of metal nanophase directly on the substrate thus allowing combining synthesis of NPs and their immobilization on the surface in a single-step procedure. The significant advantage of LID is connected with the following features (i) spatial localization of the process in the laser-affected area, (ii) no destructive effects of mild laser irradiation, as a result no decomposition of deposits or substrate, and (iii) precise control of composition, structure, and morphology of nanostructures that all together provides fine-tuning of deposits functionality.

As an object of investigation, the Pt/Au/Ag system was chosen as these metals and their combinations are of wide interest for many applications—from plasmon-enhanced phenomena to electrocatalytic processes. The chosen synthetic approach is fundamentally different from traditional methods of wet chemistry. LID is based on a photo-induced process of decomposition of organometallic precursors with the following processes of metals reduction, growing seeds, and nanospecies formation [29,30]. Therefore, the LID process provides unique conditions when the reaction system is not subjected to heating or the effect of additional chemical agents. In our previous experiments, we reported the successful formation of bimetallic Au-Ag and Pt-Ag nanoalloys in the structure of carbon nanoparticles [28,31,32,33]. However, we used specially synthesized supramolecular complexes consisting of hetero-metallic cluster core (Au-Ag or Pt-Ag accordingly) accommodated into a carbon-rich ligand environment for all these experiments. That is why the nanoalloy nature of the metal phase in the synthesized NPs was predetermined by the hetero-metallic cluster core of the precursor.

Current work deals with LID from commercially available organometallic precursors containing single metal. Multimetallic nanostructures were synthesized from mixtures of solutions of monometallic precursors. It provides fundamentally different conditions of NPs formation in comparison with LID from hetero-metallic supramolecular complexes. The current research is of interest for: (i) the understanding of multicomponent nanophase formation from a monometallic precursor, and (ii) as a simple and efficient approach for obtaining multimetallic functional nanocoatings for different applications. This is the first demonstration of multimetallic nanostructures obtained as a result of LID from commercially available organometallic precursors containing single metal. Mono-, bi- and tri-metallic nanoparticles were obtained as a result of single step LID synthesis from monometallic precursors and characterized with SEM, TEM, EDX, XPS. A comprehensive analysis of multi-metallic nanospecies with the accent on phase composition was carried out. Mechanism of multicomponent nanophase formation in course of the LID process was suggested taking into account the peculiarities of synthesis.

## 2. Materials and Methods

### 2.1. Materials and Reagents

Double-distilled water and methanol (>99%) were used as solvents for solutions preparation. Cover glasses (Levenhuk G100 cover slips, 170 µm) and quartz glasses (optical transmission coefficient >96% for 270–2700 nm; 0.15 mm thickness) were used as substrates for LID. Two silver-containing precursors: silver benzoate hydrate (C_7_H_5_AgO_2_) and silver acetate (CH_3_COOAg); two gold-containing precursors: chloroauric acid (H[AuCl_4_]·nH_2_O) and gold triacetate (C_6_H_9_AuO_6_); and one platinum-containing precursor: tetraammineplatinum(II) hydroxide hydrate (Pt(NH_3_)_4_(OH)_2_·xH_2_O), were used as a source of metals used. Precursors were purchased from Alfa Aesar (Ward Hill, MA, USA) and used as received.

### 2.2. Laser-Induced Deposition

The precursors’ concentrations in the solutions are presented in Table S1. Dissolution process was accompanied by ultrasonic treatment for 5 min and subsequent centrifugation at 12,000 rpm for 3 min in a Sigma 2-16P (Sigma Laborzentrifugen, Osterode am Harz, Germany). All the prepared solutions were used for laser-induced deposition of monometallic (Au, Pt and Ag) systems, bimetallic (Pt-Ag, Pt-Au and Au-Ag) systems and trimetallic Pt-Ag-Au system of particles. Bimetallic Au-Ag system was prepared from methanol solutions of CH_3_COOAg + C_6_H_9_AuO_6_ and water solutions C_7_H_5_AgO_2_ + H[AuCl_4_]·nH_2_O. Bimetallic Au-Pt and Pt-Ag systems were prepared from water solutions of H[AuCl_4_]·nH_2_O + Pt(NH_3_)_4_(OH)_2_·xH_2_O and C_7_H_5_AgO_2_ + Pt(NH_3_)_4_(OH)_2_·xH_2_O. The trimetallic system was deposited from mixed water solutions of C_7_H_5_AgO_2_, H[AuCl_4_]·nH_2_O and Pt(NH_3_)_4_(OH)_2_·xH_2_O. Water is the optimal solvent for all three precursors in terms of solubility. However, the solubility of silver acetate in water is negligible, so we selected methanol for deposition of Au-Ag system from acetates. The absorption spectra of all the reacted solutions are presented in Supplementary Materials (Figure S1). The laser wavelength for the LID process was chosen in accordance with the characteristic absorption bands of the studied precursors. That is why a solid-state, continuous wave, single frequency, deep-UV laser system Coherent MBD266 (Santa Clara, CA, USA) (wavelength 266 nm, power 60 mW, unfocused laser beam, laser spot diameter 2 mm) was used as a radiation source for LID realization. The laser parameters were kept the same for all LID experiments.

**Figure 1:**
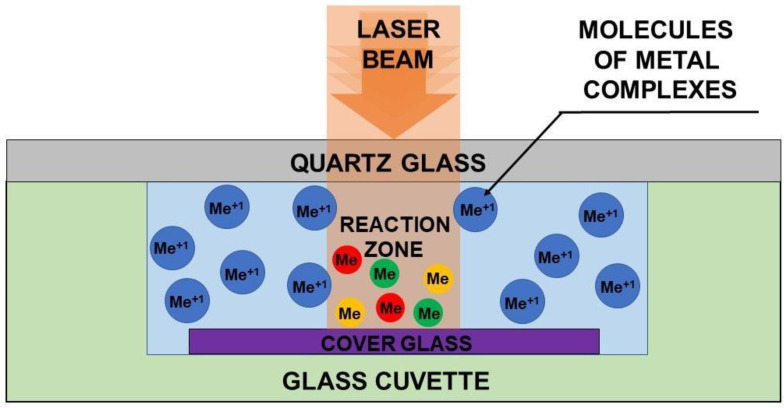
Reaction system of LID for the synthesis of metallic nanoparticles.

The scheme of the LID process is presented in Figure 1. Unfocused laser beam was directed to the substrate-solution interface through the solution. The volume of the cuvette was 80 µL; the thickness of the solution was 1 mm. Cuvette with the solution was covered with quartz glass to avoid evaporation of the solution during the LID process. LID was carried out in a stationary regime—no shift of laser beam relative to the substrate. Laser irradiation time was 40 min in all the experiments. In the case of platinum-containing systems (Pt, Ag-Pt, Au-Pt and Au-Ag-Pt) as a substrate for LID process quartz slides were used. Deposition from silver and gold complexes was performed on Menzel Microscope Coverslips only. After the LID process, the substrates were washed with isopropanol and dried at ambient conditions.

### 2.3. Characterization

The samples with deposited nanostructures were examined with scanning electron microscopy (Zeiss Merlin (Oberkochen, Germany)) and transmission electron microscopy (Zeiss Libra (Oberkochen, Germany)). The chemical composition of the samples was analyzed by means of X-ray microanalysis (Oxford Instruments INCA (Abingdon, Oxfordshire, UK)). Particles size distributions were analyzed using ImageJ software (version 1.52u, Wayne Rasband (NIH)). The XPS measurements were conducted using the photoelectron spectrometer “Escalab 250Xi” (Waltham, MA, USA) with AlKα radiation (photon energy 1486.6 eV). Spectra were recorded in the constant pass energy mode at 100 eV for survey spectrum and 50 eV for element core level spectrum, using XPS spot size 650 μm. The total energy resolution of the experiment was about 0.2 eV. For the binding energy scale calibration, the Au 4f7/2 line (84.0 eV) and the Cu 2p3/2 line (932.7 eV) were measured on the reference sample. Investigations were carried out at room temperature in an ultrahigh vacuum of the order of 1 × 10^−9^ mbar. Since the samples were deposited on a non-conductive glass substrate, an ion-electronic charge compensation system was used to neutralize the charge. Absorption spectra of organometallic precursors solutions and NPs were measured with SHIMADZU UV-2550 (Kyoto, Japan) over the spectral range 200–700 nm. The absorption spectra of metallic NPs were recorded with a Lambda 1050 Perkin Elmer (Waltham, MA, USA) in the range of 200–700 nm with an integrating Ulbricht sphere.

## 3. Results and Discussion

### 3.1. SEM Results of Mono- and Bi-Metallic Systems

**Figure 2:**
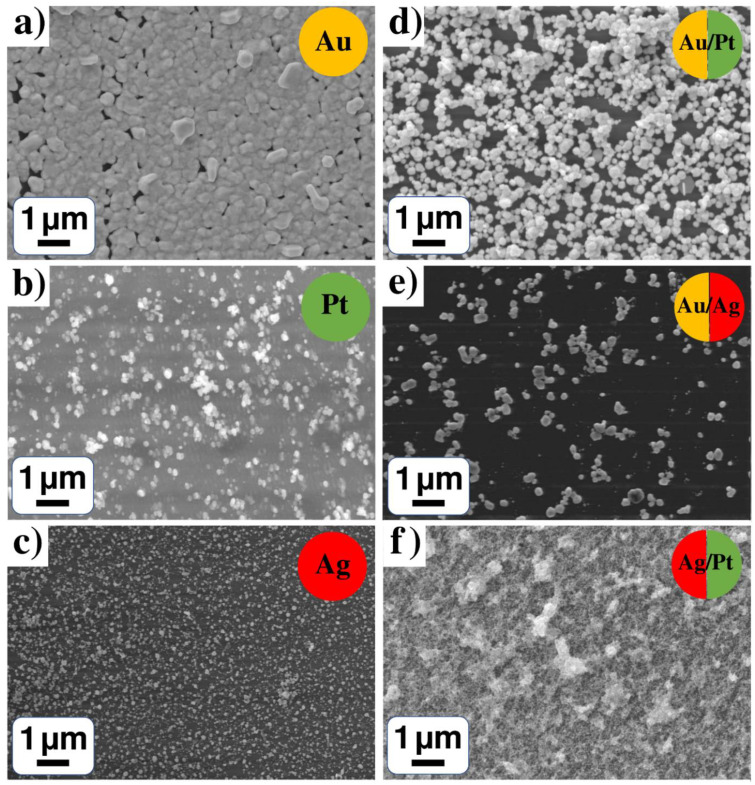
SEM results for (**a**–**c**) monometallic (Au, Pt and Ag) and (**d**–**f**) bimetallic (Pt-Ag, Pt-Au, Au-Ag) systems from water solutions: (**a**) H[AuCl_4_]·nH_2_O; (**b**) Pt(NH_3_)_4_(OH)_2_·xH_2_O; (**c**) C_7_H_5_AgO_2_; (**d**) H[AuCl_4_]·nH_2_O + Pt(NH_3_)_4_(OH)_2_·xH_2_O; (**e**) H[AuCl_4_]·nH_2_O + C_7_H_5_AgO_2_; (**f**) C_7_H_5_AgO_2_ + Pt(NH_3_)_4_(OH)_2_·xH_2_O.

Scanning electron microscopy (SEM) confirmed the successful formation of nanoparticles and conglomerates of nanoparticles on the surface of all the samples of single (Au, Pt and Ag) and binary (Pt-Ag, Pt-Au and Au-Ag) systems from water solutions (Figure 2). Despite similar experimental parameters of the LID process, one can observe different surface morphology for monometallic nanoparticles—gold particles form the densest coating in comparison with platinum and silver nanoparticles. It is interesting to note that monometallic systems are characterized by higher particle agglomeration than bimetallic structures, however, bimetallic systems result in the formation of denser films as compared with monometallic structures. It can testify to a more efficient process of laser-induced deposition for the mixed solutions because of metals interaction or their mutual influence.

Analyzing SEM images of all samples, size distributions of the observed particles and their conglomerates were obtained. The following sizes were found for single metal systems in water solutions: (Figure 2a) gold nanoparticles with a mean diameter (MD) of 215 nm and root mean square deviation (RMSD) of 230 nm; (Figure 2b) platinum nanoparticles with MD of 135 nm and RMSD of 95 nm; (Figure 2c) silver nanoparticles with MD of 57 nm and RMSD of 60 nm. Binary systems in water solutions have the following sizes: (Figure 2d) gold/platinum conglomerates with MD of 203 nm and RMSD of 155 nm; (Figure 2e) gold/silver particles with MD of 166 nm and RMSD of 174 nm. Silver/platinum conglomerates (Figure 2f) cover the surface densely, and only a rough estimation of their size can be performed, which gives MD of 25 nm and RMSD of 17 nm. The statistics of sizes for NPs of single (Au, Pt and Ag) and binary (Pt-Ag, Pt-Au and Au-Ag) systems deposited from water solutions of precursors is summarized in Table 1. In accordance with EDX analysis, all the NPs deposited from water solutions of individual precursors C_7_H_5_AgO_2_, Pt(NH3)_4_(OH)_2_·xH_2_O, H[AuCl_4_] consist of corresponding metal (Figure S2).

**Figure 3:**
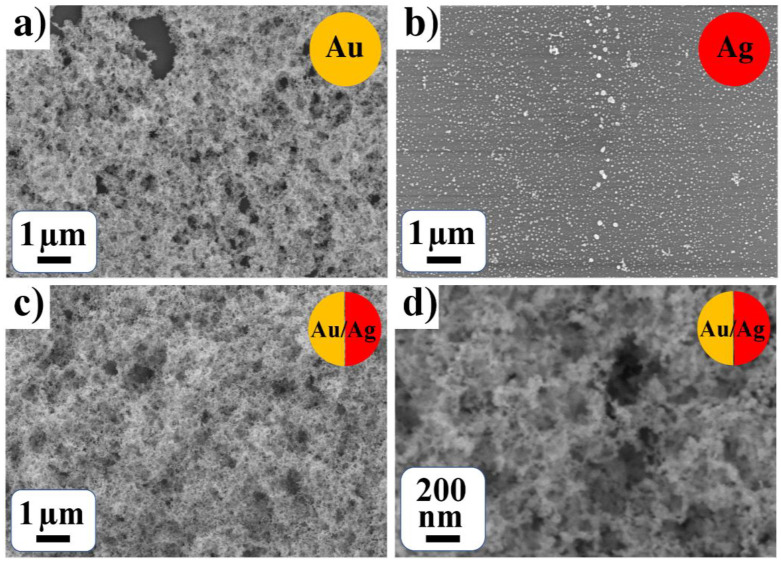
(**a**,**b**) Monometallic (Au and Ag) and (**c**,**d**) bimetallic (Au-Ag) system from methanol solutions of CH_3_COOAg and C_6_H_9_AuO_6_.

In the case of gold precursor C_6_H_9_AuO_6_ in methanol solution, gold conglomerates densely cover the surface (Figure 3a); an estimation of the sizes gives MD of 22 nm and RMSD of 14 nm (Table 1). Silver nanoparticles with MD of 49 nm and RMSD of 50 nm were observed for silver-containing precursor in methanol solution (Figure 3b). For a binary system in methanol solution, the gold/silver conglomerates cover the surface and estimation gives particle MD of 32 nm and RMSD of 22 nm (Figure 3c,d). As Pt precursor Pt(NH_3_)_4_(OH)_2_·xH_2_O is not dissolved in methanol, Pt-containing mono, bi- and tri-metallic systems in methanol were not obtained.

A comparison of Figure 2a,c,e and Figure 3a–c demonstrates the strong effect of gold precursor and solvent on the morphology for both monometallic Au and bimetallic Au-Ag nanoparticles; gold-containing deposits demonstrate the formation of continuous coatings. However, the morphology of Ag NPs in water and methanol solutions are similar and can be presented as isolated conglomerates of NPs. The observed peculiarities of the LID process are of importance for NPs creation with necessary morphology.

### 3.2. TEM and EDX Results of Bimetallic Particles Systems

#### 3.2.1. Bimetallic (Ag-Pt) System from Water Solutions

**Figure 4:**
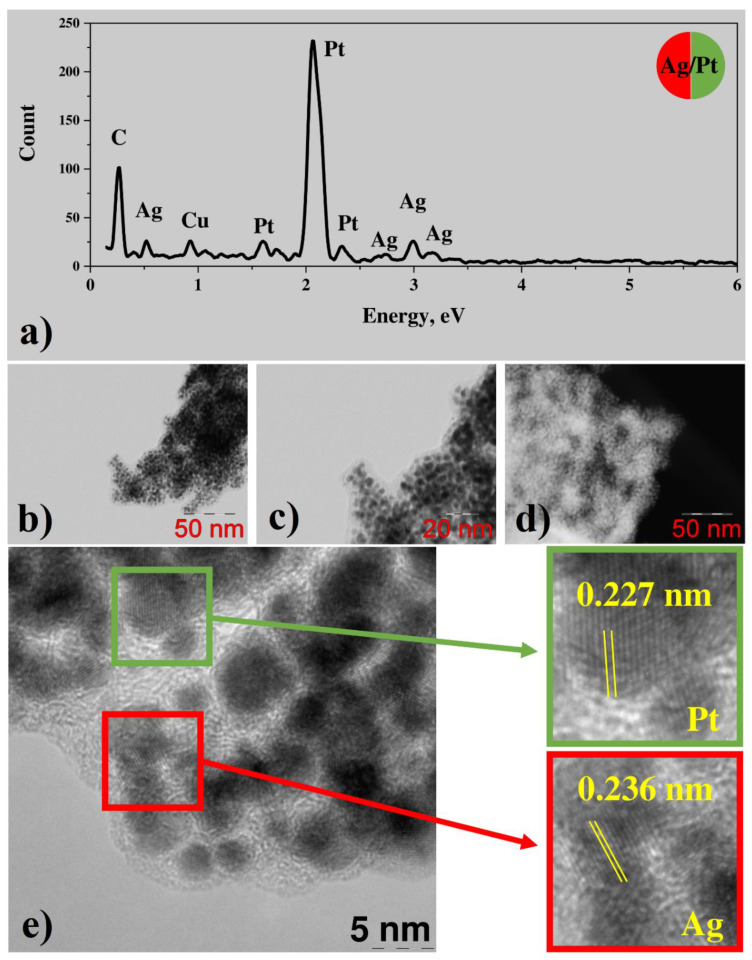
(**a**) EDX spectrum; (**b**–**d**) BF and HAADF STEM images; (**e**) HRTEM and enlarged image fragments (red: Ag; green: Pt) with areas of crystal lattice for Pt/Ag system from C_7_H_5_AgO_2_ + Pt(NH_3_)_4_(OH)_2_·xH_2_O.

To study the peculiarities of morphology and phase composition for bimetallic systems the transmission electron microscopy in various regimes and EDX analysis were applied. According to EDX, the silver/platinum nanoparticle conglomerates contain both platinum and silver, whereas the silver concentration is less by the order of magnitude than platinum concentration over the sample (Figure 4a). Besides metals, some amount of carbon is observed in the sample. The weak copper peak can be attributed to the TEM specimen grid here and further in all EDX spectra presented in this work. Investigation with STEM (Figure 4b–d) shows that Pt/Ag conglomerates consist of nanoparticles with the size of 4–5 nanometers in a light matrix that is presumably carbonaceous in accordance with EDX analysis. It is interesting to note that in the case of laser-induced deposition of monometallic NPs, the average size is ca. 50 nm for Ag nanoclusters, and 4–5 for Pt (STEM results presented in Figures S5 and S6). High-resolution TEM (HRTEM) images (Figure 4e) reveal prominent lattice fringes, indicating the crystallinity of the nanoparticles. According to Fast Fourier transform (FFT) analysis of HRTEM images, most of the fringes have a period of about 0.227 nm, corresponding to (111) planes of platinum (Figure 4e). However, some of the nanoparticles demonstrate fringes with the period of 0.236 nm, corresponding to (111) planes of silver (Figure 4e), thus indicating the presence of separate crystalline areas of platinum and silver. A weak FFT signal, corresponding to the period in between periods of silver and platinum is observed as well. Thus, lattice distortion due to alloy formation in the contact of two metals can be assumed.

#### 3.2.2. Bimetallic (Au-Pt) System from Water Solutions

**Figure 5:**
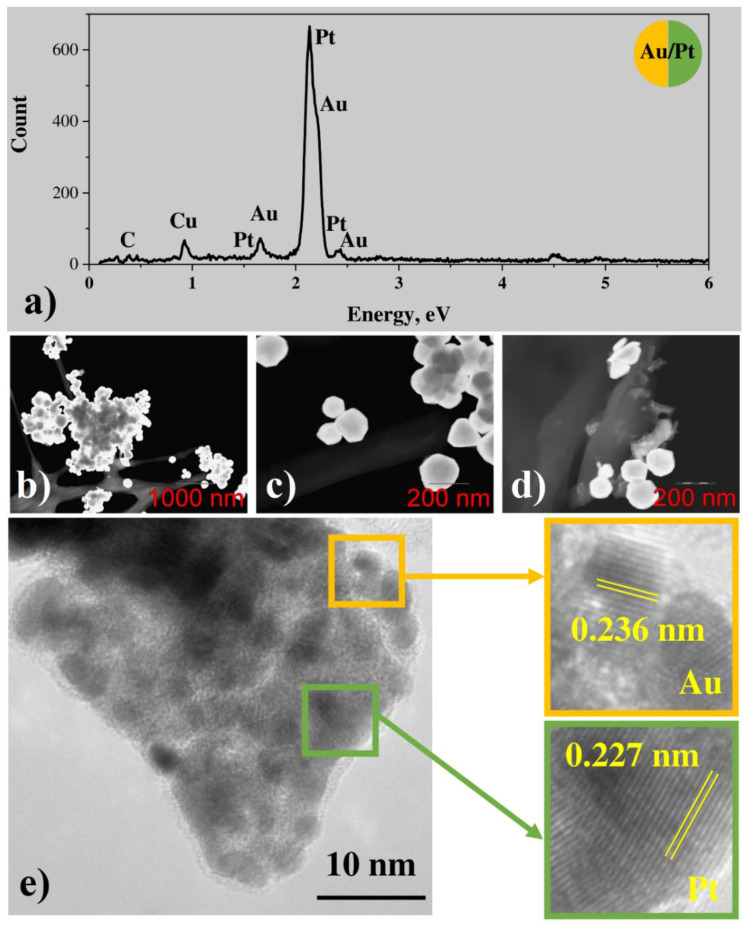
(**a**) EDX spectrum; (**b**–**d**) BF and HAADF STEM images; (**e**) HRTEM result and enlarged image fragments (orange: Au; green: Pt) with areas of crystal lattice for Pt/Au system from H[AuCl_4_]·nH_2_O + Pt(NH_3_)_4_(OH)_2_·xH_2_O.

In the case of the Au-Pt system, EDX demonstrates the presence of both gold and platinum, and the concentration of platinum is less than the one of gold by more than an order of magnitude (Figure 5a). A low carbon peak is observed in the EDX spectrum as well. STEM and TEM images show the presence of both large gold particles with the size of hundreds of nm (Figure 5b–d) and conglomerates of small particles with the size of several nanometers (Figure 5e). While STEM results for monometallic gold nanoparticles demonstrate big crystallites only with an average size of about 100–200 nm (Figure S4). Prominent lattice fringes are observed in the TEM image of small nanoparticles. FFT-analysis shows that most of the nanoparticles demonstrate fringes with a period of about 0.236 A, corresponding to the interplanar spacing of Au (111) planes. However, few particles demonstrate the period of about 0.227 nm, corresponding to (111) planes of Pt (Figure 5e). Thus, small crystalline platinum nanoparticles are present among small and large gold particles. Analogously to the case of the silver-platinum system, some FFT signal corresponding to the period in between periods of gold and platinum is observed as well, so alloy formation due to the contact of two metals can be concluded.

#### 3.2.3. Bimetallic (Au-Ag) System from Water Solutions

**Figure 6:**
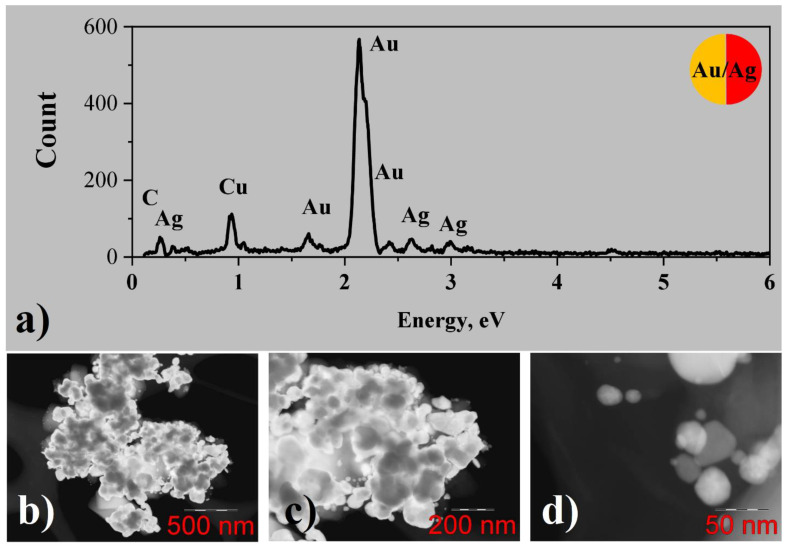
(**a**) EDX spectrum; (**b**–**d**) STEM images for Au/Ag system from water solutions of H[AuCl_4_]·nH_2_O and C_7_H_5_AgO_2_.

EDX analysis of gold/silver conglomerates from water solutions (Figure 6a) shows the presence of gold and silver, and the concentration of silver is approximately ten times less than the one of gold. Some low amounts of carbon and chlorine from precursors can also be found. The copper peak can be attributed to the TEM specimen grid. STEM images (Figure 6b–d) demonstrate that large particles with the size of tens or even hundreds of nanometers are formed, being in good agreement with data obtained from SEM measurements. Some of the particles demonstrate channeling contrast and straight faces, which indicates their crystallinity. HRTEM images were not analyzed in this case, since gold and silver have very close lattice constants of 4.078 and 4.085 A correspondingly, making their lattice fringes indistinguishable. One can suppose that in this case not only pure gold and silver nanoparticles but also the nanoparticles of bimetallic alloys can be formed.

#### 3.2.4. Bimetallic (Au-Ag) System from Methanol Solutions

**Figure 7:**
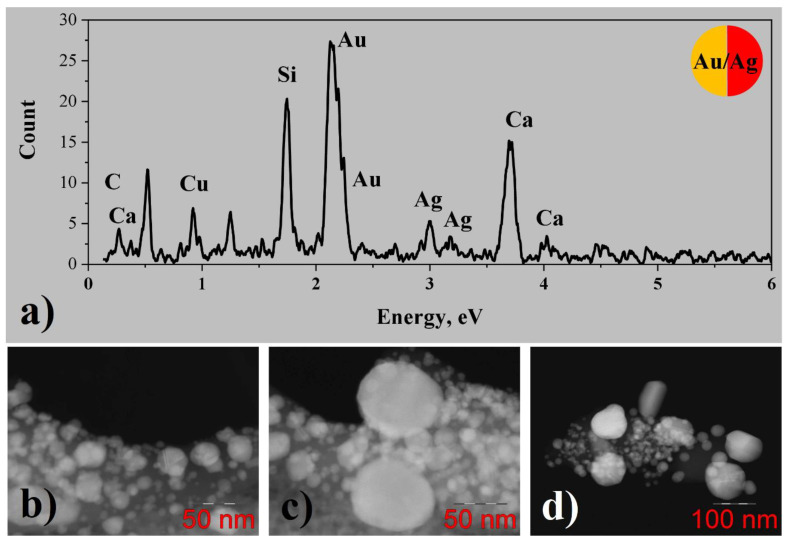
(**a**) EDX spectrum; (**b**–**d**) STEM images for Au/Ag system from methanol solutions of CH_3_COOAg and C_6_H_9_AuO_6_.

EDX analysis of gold/silver conglomerates from methanol solutions (Figure 7a) demonstrates the presence of gold and silver, and the concentration of silver is less than the one of gold, varying from 0 up to 30 atomic percent for different particles. Some amount of carbon from the precursor is observed as well. Besides that, silicon, oxygen, and calcium peaks are clearly visible in the spectrum, which can originate from contamination of the sample with some particles of glass substrate during the transfer to the TEM specimen grid.

According to STEM investigation (Figure 7b–d), gold/silver conglomerates consist of metal particles with the size of several tens of nanometers, corresponding to the one obtained from the SEM image, connected by means of a carbonaceous matrix. Some of the particles reveal diffraction contrasts indicating their crystallinity. HRTEM images were not obtained for such large particles, but in this case, again HRTEM would not give new information, since gold and silver have very close lattice constants. Similarly, in the case of the gold/silver conglomerates from water solution, one can assume that in this case not only pure gold and silver nanoparticles but the nanoparticles of bimetallic alloys can be formed as well.

On a basis of analysis of TEM, STEM, FFT results we can conclude that in all cases of bimetallic systems the crystalline metallic nanoparticles are formed. In most cases, it is a mixture of nanoparticles of single metals (Pt and Ag, Pt and Au). For the gold-silver system, the phase distinguishing (monometallic or alloy) is problematic as the lattice parameters of these two metals are close to each other. One can note that pure gold and silver tend to form particles with the size up to hundreds of nanometers, whereas platinum particles are conglomerates of small nanoparticles ca 2–3 nm. In the case of Au-Ag and Au-Pt systems, the large (ca 200 nm) as well as small (3–5 nm) gold nanoparticles are observed. Since the concentration of gold precursor exceeds the concentration of another metal (silver or platinum), we can suppose that in the case of bimetallic solutions the large gold particles are formed similarly to the case of the monometallic gold system. Whereas smaller Au NPs are formed in the vicinity of another metal, thus indicating the mutual effect of metals in the process of particle growth.

### 3.3. SEM, TEM and EDX Results of Trimetallic Particles Systems

**Figure 8:**
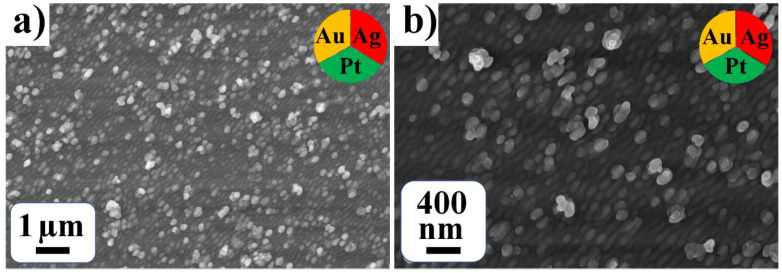
SEM images with different resolutions (**a**,**b**) for trimetallic Pt-Ag-Au system from water solutions.

SEM images of the trimetallic system in water solution (Figure 8) show the formation of a dense film of nanoparticles and their conglomerates. An estimation gives the mean diameter of the conglomerates of 158 nm (Table 1).

**Figure 9:**
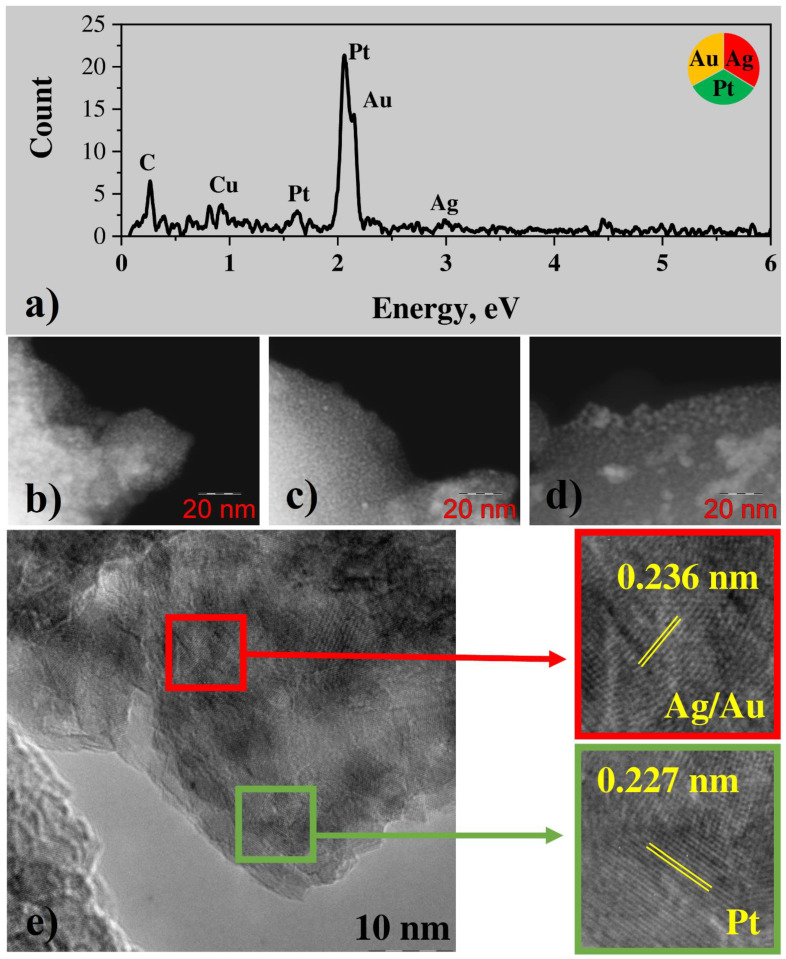
(**a**) EDX spectrum; (**b**–**d**) STEM (HAADF) images; (**e**) HRTEM result and enlarged image fragments (red: Ag/Au; green: Pt) with areas of crystal lattice for triple Pt-Ag-Au system from water solutions.

EDX analysis of the conglomerates (Figure 9a) shows that they consist mostly of platinum. Gold and silver are also present in the sample, but their amount is very low. Besides metals, some amount of carbon from precursor residuals is observed in the sample as well. STEM images of trimetallic conglomerates (Figure 9b–d) show that they consist of metal nanoparticles with the diameter of a few nanometers, connected with a carbonaceous matrix. HRTEM images of the conglomerates (Figure 9e) demonstrate lattice fringes, indicating the crystallinity of the nanoparticles. FFT-analysis shows that some of the particles have a lattice period of 0.236 nm (Figure 9e), which can be attributed to (111) planes of gold or silver, or gold/silver alloy. However, most of the fringes have the period of 0.227 nm, which corresponds to platinum (111) planes, thus indicating that in this case most of the nanoparticles are platinum ones; while separate gold, silver, or gold/silver nanoparticles can be also found.

**Figure 10:**
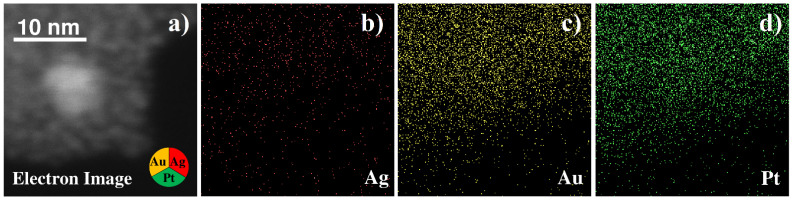
TEM image (**a**) and EDX elemental mapping analysis (**b**–**d**) of triple Pt-Ag-Au system from water solutions.

EDX maps of silver, gold and platinum show uniform distribution of these elements across the sample (Figure 10), and individual metal nanoparticles cannot be observed because of limitations of this experiment, because of unguided sample drift during prolonged map acquisition.

### 3.4. XPS Results

**Figure 11:**
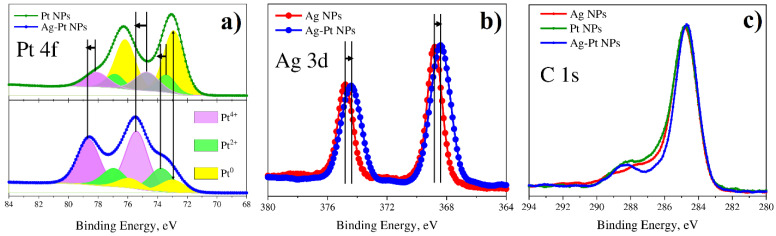
XPS results for monometallic (Pt and Ag) and bimetallic (Pt-Ag) systems of NPs synthesized from water solutions: (**a**) Pt 4f; (**b**) Ag 3d; (**c**) C 1s.

**Figure 12:**
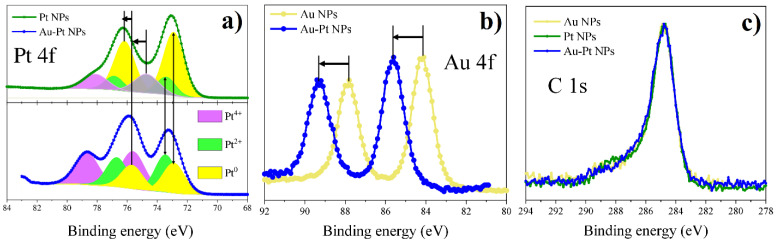
XPS results for monometallic (Pt and Au) and bimetallic (Pt-Au) systems of NPs synthesized from water solutions: (**a**) Pt 4f; (**b**) Au 4f; (**c**) C 1s.

**Figure 13:**
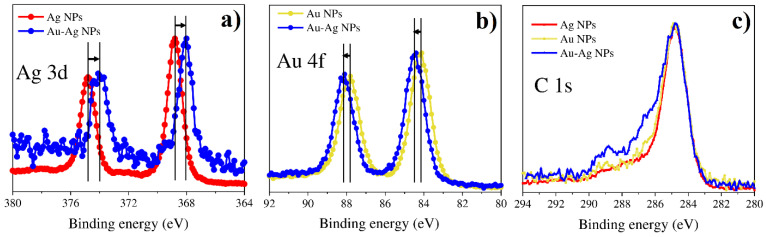
XPS results for monometallic (Au and Ag) and bimetallic (Au-Ag) systems of NPs synthesized from water solutions: (**a**) Ag 3d; (**b**) Au 4f; (**c**) C 1s.

**Figure 14:**
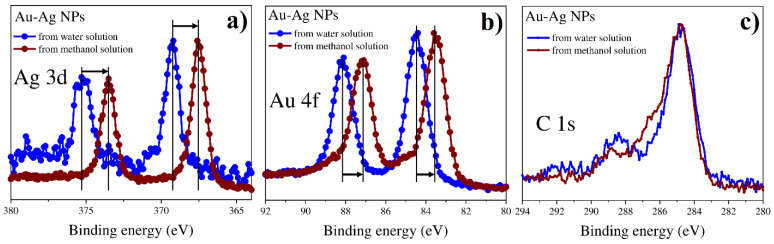
XPS results for bimetallic (Au-Ag) systems of NPs synthesized from water and methanol solutions: (**a**) Ag 3d; (**b**) Au 4f; (**c**) C 1s.

**Figure 15:**
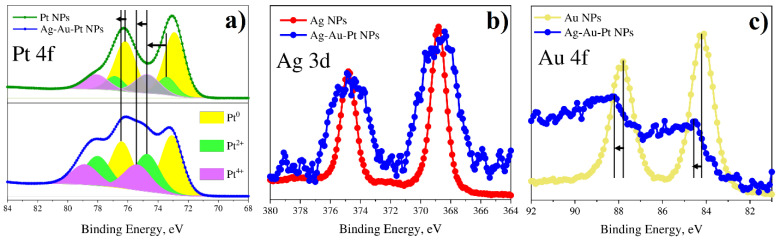
XPS results for single (Pt, Ag and Au) and triple (Pt-Ag-Au) systems of NPs synthesized from water solutions: (**a**) Pt 4f; (**b**) Ag 3d; (**c**) Au 4f.

The XPS method was used as a tool to study the peculiarity of multimetallic nanoparticles and identify metal phases for bi- and tri-metallic systems. Figure 11, Figure 12, Figure 13 and Figure 14 show the spectra for Ag/Pt, Au/Pt, and Ag/Au binary samples in comparison with the spectra of monometallic Ag, Au, and Pt nanoparticles obtained from the same precursors. Figure 15 shows XPS spectra for a system with three metals (Au-Ag-Pt).

The shifts of the main lines by energy are presented in Table 2 for all XPS spectra. It is important to note that the presence of carbon was detected in all the cases. The position of the C-C bond for the C 1s peak for all samples was found to be 284.8 ± 0.1 eV (Figure 11, Figure 12, Figure 13 and Figure 14 and Figure S3) and testifies towards carbon of sp^3^ hybridization.

In all the observed XPS spectra one can detect energy shifts for metals in bi- or tri-metallic systems in comparison with line positions of single metals. The shifts can be connected with changes in particle size or electron density redistribution due to alloy formation.

#### 3.4.1. Bimetallic (Ag-Pt) System from Water Solutions

Figure 11 shows Pt 4f, Ag 3d and C 1s spectra for a binary Ag-Pt system in comparison with single Ag and Pt nanoparticles. The Pt 4f spectrum of single Pt nanoparticles, in addition to the main metallic peak Pt^0^, has components with higher binding energies that can be associated with the oxidation states of platinum 2+ and 4+. However, for the Pt 4f spectrum of the binary Ag-Pt system, the relative intensity of these components (Pt^2+^, Pt^4+^) are much larger and have higher binding energy compared to single metal nanoparticles. At the same time, the Ag 3d peak is shifted towards lower binding energies as compared to Ag nanoparticles. Thus, it testifies a redistribution of electrons between the 4f states of platinum and 3d states of silver in favor of the latter. This indicates the formation of a bimetallic alloy in the case of Ag-Pt particles.

#### 3.4.2. Bimetallic (Au-Pt) System from Water Solutions

Figure 12 shows Pt 4f, Au 4f and C 1s spectra for a bimetallic Au-Pt system in comparison with monometallic Au and Pt nanoparticles. Platinum in Au-Pt nanoparticles is characterized by behavior similar to platinum in Ag-Pt sample. In the Pt 4f spectrum, the peaks of the 2+ and 4+ states also have a high intensity. However, in contrast to the Pt 4f spectrum of Ag-Pt nanoparticles, the energies of the platinum oxidation states 0 and 2+ have lower binding energy. In this case, the binding energy is close to that of monometallic Pt nanoparticles. At the same time, the binding energy of the Au 4f peak is higher than for Au nanoparticles. It can be assumed that in this case there is a redistribution of electrons in favor of platinum. This also indicates the emergence of a bimetallic alloy of platinum and gold.

#### 3.4.3. Bimetallic (Au-Ag) Systems from Water and Methanol Solutions

XPS spectra for bimetallic Ag-Au particles obtained from different solutions are shown in Figure 13 and Figure 14, respectively. Figure 13 demonstrates Ag 3d, Au 4f, and C 1s spectra for a bimetallic Ag-Au system in water solution in comparison with individual Ag and Au nanoparticles. For the case of a binary system, the peak Au 4f is shifted towards higher binding energies as compared to single Au nanoparticles, while the peak Ag 3d is shifted towards lower binding energies as compared to single Ag nanoparticles. This indicates the formation of an alloy in the Ag-Au nanoparticles. At the same time, it is interesting to note the significant shift of binding energy of both Ag and Au to the lower region for Ag-Au nanoparticles obtained from methanol solutions in comparison with individual Ag and Au nanoparticles and Ag-Au NPs from water solutions (Figure 14). It is worth noting here that the sample obtained from the mixture of solutions in methanol consists of smaller particles according to SEM and TEM results. Thus, the dimensional effect can explain the unidirectional shift of Ag 3d and Au 4f peaks in bimetallic systems synthesized from different precursors.

#### 3.4.4. Trimetallic (Au-Ag-Pt) System from Water Solutions

Figure 15 shows the spectra of Ag 3d, Pt 4f, Au 4f, and C 1s for the triple system Au-Ag-Pt in comparison with single Ag, Au and Pt nanoparticles. The components of the Pt 4f spectrum and the Au 4f line for the ternary compound are shifted towards higher binding energies, while the binding energy of silver practically coincides with the single Ag sample. However, one can note the pronounced width increase of Ag 3d band for the case of trimetallic system Au-Ag-Pt in comparison with single Ag NPs. The obtained results testify to the mutual effect of components in the Au-Ag-Pt system resulting in obviously shifted B.E. because of the change of chemical environment of the metals Ag, Au, Pt and the electron transfer effect between them. All in all, the XPS results verify the formation of alloy phase in the composition of multimetallic nanoparticles that is consistent with the STEM, FFT-analysis and HRTEM results.

### 3.5. Absorption Spectra of Particles Systems

**Figure 16:**
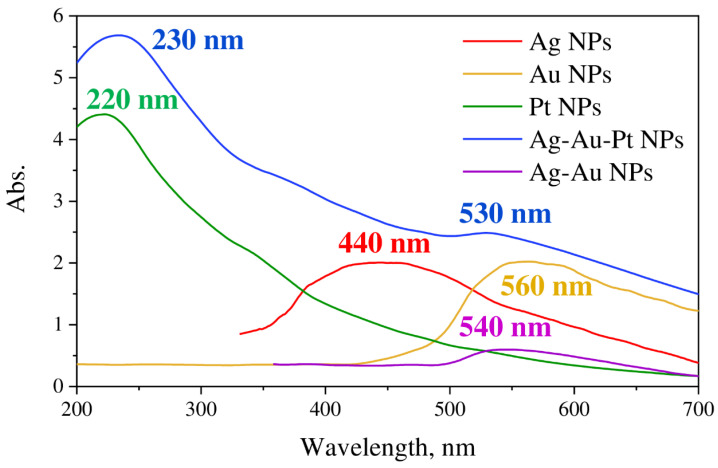
Absorption spectra of monometallic (Ag, Au and Pt), bimetallic Au-Ag and trimetallic Au-Ag-Pt NPs.

Absorption spectra of mono- (Ag, Au and Pt) and tri-metallic Au-Ag-Pt NPs measured in the spectral range of 200–700 nm are shown in Figure 16. One can see that monometallic NPs demonstrated characteristic peaks corresponding to localized surface plasmon resonance. Positions of the observed LSPR peaks are 440, 560, and 220 nm for Ag, Au, and Pt NPs, correspondingly, which coincide with previously reported literature data [34,35,36]. The absorption spectrum of trimetallic Au-Ag-Pt NPs displayed two separated LSPR peaks with maxima at 230 and 530 nm. Noteworthy, these peaks are close but do not coincide with the position of LSPR for monometallic Pt and Au NPs. The shifts demonstrate the mutual influence of metals in the Au-Ag-Pt system. The red shift of the Pt NPs SPR is caused by the presence of silver and gold in the structure. Ag NPs result in the blue shift of AuNPs SPR [23].

The absorption spectrum of bimetallic Ag-Au NPs showed LSPR peak at 540 nm, which is located between the position of the peaks for monometallic Ag and Au NPs (Figure 16). It should be noted that relative to the trimetallic system a small peak shift is also observed. This may be due to the different concentration ratios of gold and silver in the systems as well as the effect of platinum in the trimetallic system. Observed changes in the absorption spectrum of monometallic nanoparticles—LSPR shifts when introducing other metals into the system—are in good agreement with the described structures of the obtained bi- and trimetallic systems.

Based on the complex analysis of the results obtained with different techniques (TEM, EDX, XPS, UV-VIS absorption) we can conclude that in the case of all binary systems (Pt-Ag, Pt-Au, and Au-Ag), the formation of overlapping crystallites of individual metals takes place together with the formation of bimetallic alloys in the area of contact of monometallic phases. The monometallic crystallites of binary systems are characterized by (111) planes for any metal (Pt, Ag or Au). The size of crystallite was found to be ca 2–5 nm regardless of metal with the only exception for the Au-containing binary system that is characterized by the bimodal size distribution of Au nanoparticles—3–5 nm and 200 nm. It is interesting to note that monometallic nanoparticles obtained in the course of LID from precursors of individual metals are characterized by bigger sizes from 140 to 200 nm in the case of Au (Figure S4), 40–60 nm for Ag (Figure S5), while Pt NPs were found to be 2–3 nm (Figure S6). In the case of the triple Pt-Ag-Au system, one can also see the formation of individual crystallites of metals with sizes of 1.5–2 nm with some multimetallic alloy phase between the monometallic crystallites. In such a way, the obtained results demonstrate the formation of nanostructures that are combinations of monometallic crystallites with shells of multimetallic phases. The results of UV-VIS spectroscopy demonstrate LPSR peaks confirming the conclusions of structural analysis.

The formation of the monometallic crystallites surrounded by multi-metal alloy is determined by the peculiarity of the LID process. As a first step, photodecomposition of the metal precursor takes place with intramolecular redox process resulting in seeds of metal phase. Then the following growth of metal seeds occurs. If the reaction system contains precursors of various metals and their seeds, the further growth of monometallic nanocrystallites leads to the aggregation process with the formation of an alloy phase between dissimilar nanocrystallites. If the system contains single metal, the nanocrystallites aggregation results in nanoparticles of bigger sizes. The observed sizes distributions of LID deposited NPs for monometallic and multimetallic systems confirm the suggested particle growth process.

## 4. Conclusions

Mono- and multimetallic nanoparticles of various combinations of Au, Ag, Pt metals were successfully synthesized by laser-induced deposition from water and methanol solutions of commercially available precursors. Nanoparticles and conglomerates of nanoparticles form more or less dense and continuous coatings on the surface of the samples. The size of monometallic nanoparticles was found to be dependent on the metal—140–200 nm for Au, 40–60 nm for Ag, 2–3 nm for Pt under the same experimental conditions. While bi- and trimetallic nanoparticles were core-shell structures representing monometallic crystallites surrounded by an alloy of respective metals. Alloy formation was detected with FFT analysis and confirmed by XPS. HRTEM and FFT analysis showed the formation of crystallites with lattice fringes attributed to (111) for all the studied metals. The size of monometallic clusters (Ag, Au, Pt) in the structure of multimetallic nanoparticles is ca. 2–5 nm for any variant of the multimetallic system (Ag-Pt, Au-Pt, Au-Ag-Pt). The only case of bimodal size distribution with mean sizes 3 and 200 nm was detected for Au nanoclusters in the Au-Pt system that is most likely caused by gold precursor prevalence in the LID solution.

The process of NPs formation is starting with photodecomposition of the metal precursor resulting in seeds of metal and following growth of metal phase. The formation of an alloy phase takes place between monometallic nanocrystallites of different metals in course of their growth and agglomeration stage.

## Figures and Tables

**Table 1:** Statistics of sizes for metal particles and their conglomerates from SEM results.

Solvent	Parameter	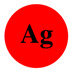	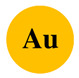	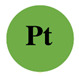	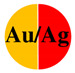	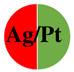	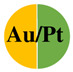	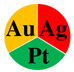
H_2_O	MD, nm	57	215	135	166	25	203	158
	RMSD, nm	60	230	95	173	17	155	68
CH_3_OH	MD, nm	49	22	-	32	-	-	-
	RMSD, nm	50	14	-	22	-	-	-

**Table 2:** Binding energy of elements for monometallic Au, Ag, Pt particles, bimetallic Au-Ag, Au-Pt, Ag-Pt and triple Au-Ag-Pt particles.

Spectrum Name	Ag BE, eV	Au BE, eV	Pt BE, eV	Au-Ag BE, eV	Ag-Pt BE, eV	Au-Pt BE, eV	Au-Ag-Pt BE, eV
C 1s	284.79	284.81	284.79	284.79	284.79	284.82	284.77
Ag 3d	368.80	-	-	368.03	368.50	-	368.75
Au 4f	-	84.18	-	84.46	-	84.71	84.59
Pt 4f7 0	-	-	72.91	-	73.40	73.21	73.08
Pt 4f7 2+	-	-	73.40	-	75.29	74.46	74.72
Pt 4f7 4+	-	-	74.73	-	76.95	75.45	75.38

## Data Availability

The data presented in this study are available on request from the corresponding author.

## Supplementary Materials

The following supporting information can be downloaded at: https://www.mdpi.com/article/10.3390/nano12010146/s1, Table S1: Concentrations (mM) of metal precursors in solutions used for LID; Figure S1. Absorption spectra of complexes solutions (a) in water and (b) in methanol; Figure S2. EDX spectrum for single NPs systems from water solutions: (a) C_7_H_5_AgO_2_; (b) Pt(NH_3_)_4_(OH)_2_·xH_2_O; (c) H[AuCl_4_]·nH_2_O; Figure S3. C 1s XPS spectra for single (Pt, Ag and Au) and triple (Pt-Ag-Au) systems from water solutions C_7_H_5_AgO_2_ + Pt(NH_3_)_4_(OH)_2_·xH_2_O + H[AuCl_4_]·nH_2_O in H_2_O; Figure S4. STEM images with different resolutions (a–c) for monometallic Au particles; Figure S5. STEM images with different resolutions (a–c) for monometallic Ag particles; Figure S6. STEM images with different resolutions (a–c) for monometallic Pt particles.
