# Supplementary material for: Single Step Laser-Induced Deposition of Plasmonic Au, Ag, Pt Mono-, Bi- and Tri-Metallic Nanoparticles
Source: Nanomaterials (Basel). 2021 Dec 31;12(1):146. doi: 10.3390/nano12010146 (PMC8746481; doi:10.3390/nano12010146)
Supplement: Supplementary file 1 [file nanomaterials-12-00146-s001.zip › nanomaterials-1503356-SI.pdf]

nanomaterials

Supplementary Information

Single Step Laser-Induced Deposition of Plasmonic Au, Ag, Pt Mono-, Bi- and Tri-Metallic Nanoparticles

Daria V. Mamonova [1], Anna A. Vasileva [1], Yuri V. Petrov [2], Alexandra V. Koroleva [3], Denis V. Danilov [4], Ilya E. Kolesnikov [5], Gulia I. Bikbaeva [1], Julien Bachmann [1,6] and Alina A. Manshina [1,*]

1 Institute of Chemistry, Saint-Petersburg State University, 26 Universitetskii Prospect, Saint-Petersburg 198504, Russia; magwi@mail.ru (D.V.M.); anvsilv@gmail.com (A.A.V.); st086467@student.spbu.ru (G.I.B.); julien.bachmann@fau.de (J.B.)
2 Department of Physics, Saint-Petersburg State University, Ulyanovskaya 3, Saint-Petersburg 198504, Russia; y.petrov@spbu.ru
3 Center for Physical Methods of Surface Investigation, Research Park, Saint Petersburg University, Universitetskiy Prosp. 35, Lit. A, Saint-Petersburg 198504, Russia; koroleva.alexandra.22@gmail.com
4 Interdisciplinary Resource Center for Nanotechnology, Research Park, Saint-Petersburg State University, Ulyanovskaya 1, Saint-Petersburg 198504, Russia; danilov1denis@gmail.com
5 Centre for Optical and Laser Materials Research, Research Park, Saint-Petersburg State University, Ulyanovskaya 5, Saint-Petersburg 198504, Russia; ilya.kolesnikov@spbu.ru
6 Department of Chemistry and Pharmacy, Friedrich–Alexander University of Erlangen–Nürnberg, IZNF, Cauerstr. 3, 91058 Erlangen, Germany
* Correspondence: a.manshina@spbu.ru

Table S1. Concentrations (mM) of metal precursors in solutions used for LID.

	**Solvent →**	H_2O	CH_3OH
**Metal system**	**Precursor ↓**	**Concentration, mM**	
Ag	$C_7H_5AgO_2$	5.8	—
	CH_3COOAg	—	7.0
Au	$H[AuCl_4]\cdot nH_2O$	11.6	—
	$C_6H_9AuO_6$	—	7.5
Pt	$Pt(NH_3)_4(OH)_2\cdot xH_2O$	5.3	—
Ag-Au	$C_7H_5AgO_2$	0.7	—
	+		
	$H[AuCl_4]\cdot nH_2O$	14.5	
	CH_3COOAg	—	3.5
	+		
	$C_6H_9AuO_6$		3.7
Ag-Pt	$C_7H_5AgO_2$	1.1	—
	+		
	$Pt(NH_3)_4(OH)_2\cdot xH_2O$	2.2	
Au-Pt	$H[AuCl_4]\cdot nH_2O$	8.8	—
	+		
	$Pt(NH_3)_4(OH)_2\cdot xH_2O$	0.6	
Ag-Au-Pt	$C_7H_5AgO_2$	1.7	—
	+		
	$H[AuCl_4]\cdot nH_2O$	0.15	
	+		
	$Pt(NH_3)_4(OH)_2\cdot xH_2O$	1.0	

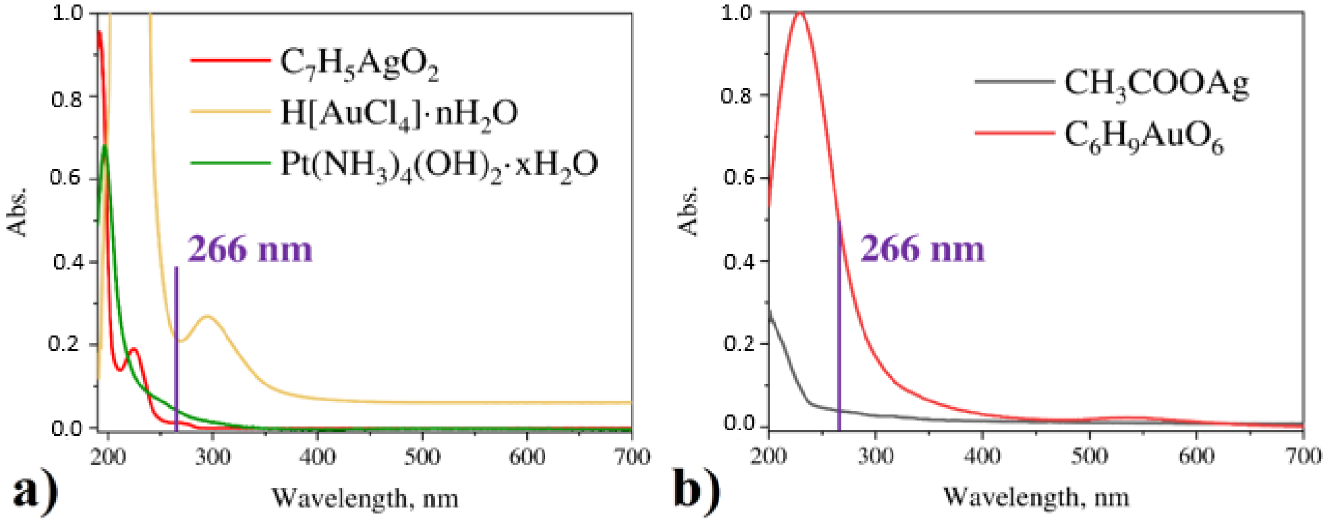

Figure S1. Absorption spectra of complexes solutions (**a**) in water and (**b**) in methanol.

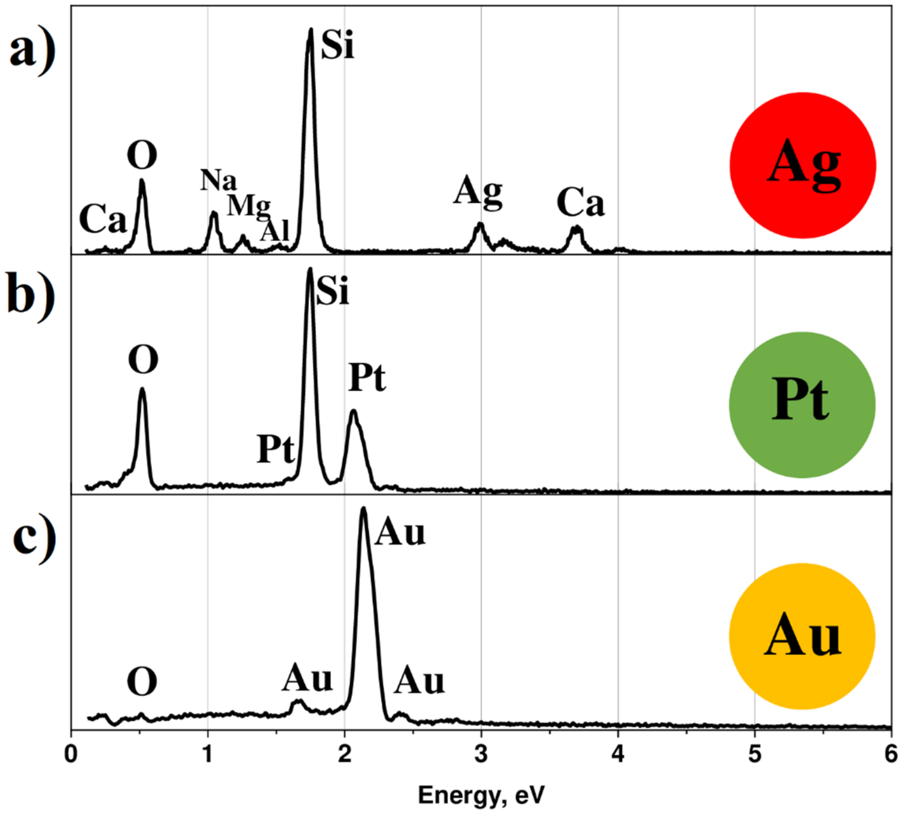

Figure S2. EDX spectrum for single NPs systems from water solutions: (**a**) $C_7H_5AgO_2$; (**b**) $Pt(NH_3)_4(OH)_2\cdot xH_2O$; (**c**) $H[AuCl_4]\cdot nH_2O$.

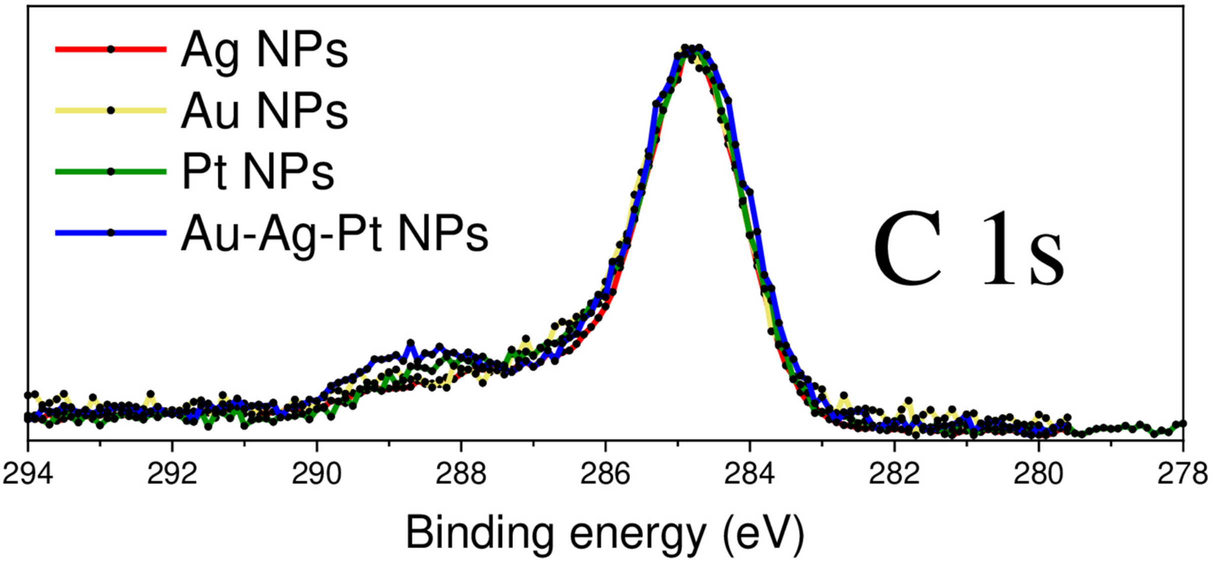

Figure S3. C1s XPS spectra for single (Pt, Ag and Au) and triple (Pt-Ag-Au) systems from water solutions $C_7H_5AgO_2$ + $Pt(NH_3)_4(OH)_2{\cdot}xH_2O$ + $H[AuCl_4]{\cdot}nH_2O$ in H_2O.

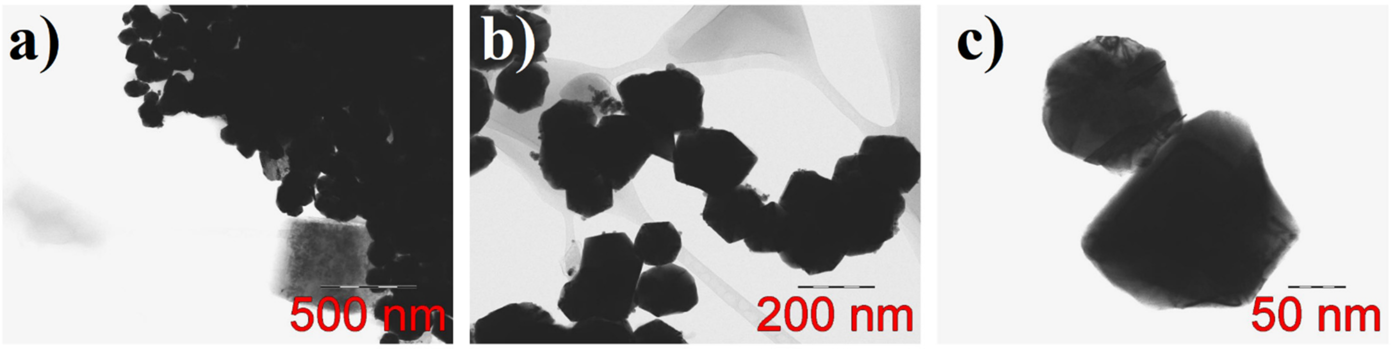

Figure S4. STEM images with different resolutions (**a**–**c**) for monometallic Au particles.

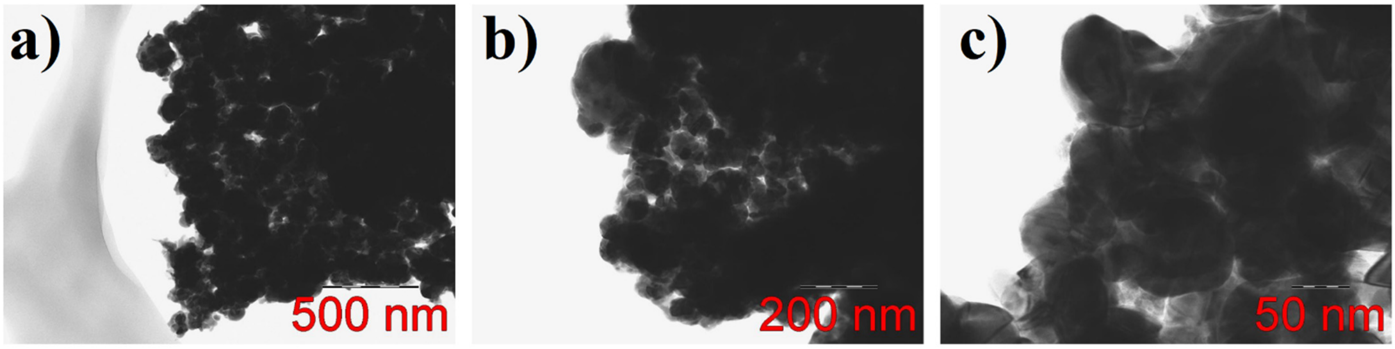

Figure S5. STEM images with different resolutions (**a**–**c**) for monometallic Ag particles.

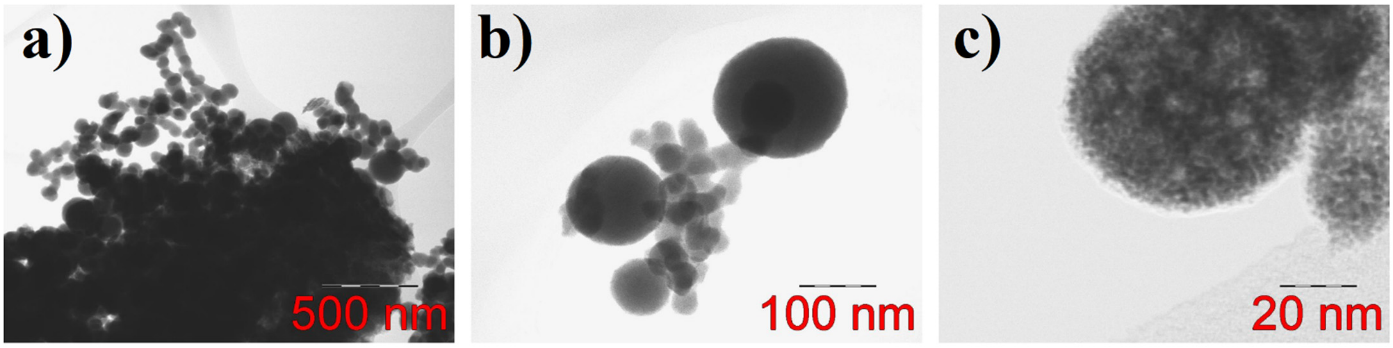

Figure S6. STEM images with different resolutions (**a**–**c**) for monometallic Pt particles.
